# Phytochemical profiling of *Livistona carinensis* leaf extract *via* UHPLC-QTOF-MS/MS with assessment of its antiviral mechanisms†

**DOI:** 10.1039/d4ra02705a

**Published:** 2024-07-05

**Authors:** Amr M. K. Mahrous, Mohamed S. Hifnawy, Rehab M. S. Ashour, Marwa Yousry Issa, Ahmed Zayed

**Affiliations:** a Department of Pharmacognosy, Faculty of Pharmacy, El Saleheya El Gadida University El Saleheya El Gadida 44813 Egypt amrmkh@gmail.com; b Department of Pharmacognosy, Faculty of Pharmacy, Cairo University Kasr El-Aini Street Cairo 11562 Egypt; c Department of Pharmacognosy, College of Pharmacy, Tanta University El-Guish Street (Medical Campus) 31527 Tanta Egypt ahmed.zayed1@pharm.tanta.edu.eg

## Abstract

Among 36 species of the genus *Livistona* (family Palmae or Arecaceae), *L. carinensis* is considered the only species native to Africa. Previous studies showed the richness of *Livistona* fruits in phenolic compounds. The goal of the current study was to investigate the phytochemical composition and assess the antiviral mechanisms of the *L. carinensis* leaves' ethanolic extract cultivated in Egypt for the first time. The ultra-high performance liquid chromatography-quadrupole time-of-flight mass spectrometry (UHPLC-QTOF-MS/MS) was applied. Moreover, the total crude extract was fractionated using ethyl acetate and *n*-butanol for phytochemical investigations by various chromatographic and spectroscopic techniques. Besides, the antiviral activity of *L. carinensis* leaves was assessed using three protocols *in vitro* using MTT assay compared to acyclovir. UHPLC-QTOF-MS/MS-based analysis resulted in identification of 72 metabolites tentatively. They belonged to diverse phytochemical classes, mainly including flavonoids (29), organic acids (10), and phenolic acids (7). The antiviral activity investigations revealed a direct Adeno virus inactivation mechanism rather than inhibition of virus replication or blocking its attachment to Vero cells. Hence, the plant leaves may be a potential candidate for discovery of novel antiviral drugs owing to the diversity of identified phytochemical classes.

## Abstract 2

Among 36 species of the genus *Livistona* (family Palmae or Arecaceae), *L. carinensis* is considered the only species native to Africa.

## Graphical abstract

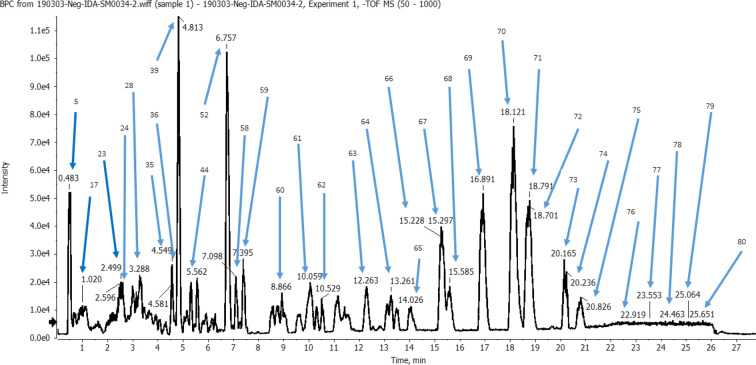

## 1. Introduction

About 2600 species of palm trees in 181 genera make up the Arecaceae family, which is primarily found in tropical and subtropical regions of the globe. Mostly, they are planted primarily for decorative, and landscape uses or are only targeted for the local and regional markets. Nevertheless, oil palm (*Elaeis guineensis* Jacq.), date palm (*Phoenix dactylifera* L.), and coconut palm (*Cocos nucifera* L.) are the most three traded crops among the economically potential palm species. Over 35% of the world's oils and fats come from oil palm and coconut, while date palm is crucial to food security and agricultural output in dry and vulnerable environments around the world.^1,2^ Genera like *Phoenix* and *Cocos* have been investigated phytochemically and biologically in previous literature.^3,4^ However, others such as *Livistona* are still unexplored, and therefore, need more attention for investigating its phytochemical composition and bioactivities.^5^

Recent studies showed the richness of *Livistona* species in phenolic compounds such as the *L. decipiens* leaves' ethanolic extract in flavonoids (*e.g.*, apigenin, luteolin, quercetin, isoorientin, schaftoside, and orientin),^5^ while the fruits of *L. chinensis* were in phenolic acids as 5-*O*-caffeoylshikimic acid and 3-*O*-caffeoylshikimic acid.^6^ Other phytochemical classes were also identified such as ceramides and glycerides in the ethanol root extract.^7^ In addition, fatty acid derivatives were identified in *L. australis* fruits (*e.g.*, palmitic acid, octacosanoic acid, juniperic acid, and heptacosane) in petroleum ether fraction.^8^ These bioactive constituents were associated with a number of health promoting benefits (*e.g.*, anti-hyperlipidemic and anti-ulcer activities),^8^ cytotoxic and antiproliferative effects against human tumor cell lines with the IC_50_ of 10–65 μM.^7,9^

Apart from this, our earlier research using GLC (gas–liquid chromatography) to analyze the lipid fraction of the pericarp and seeds of *L. carinensis* fruits showed that stigmasterol was the predominant phytosterol in the pericarp and seeds, accounting for 9.0% and 10.8% of the total, respectively. Additionally, palmitic acid was the major fatty acid in the pericarp (35.0%). Moreover, linoleic acid was the major among unsaturated fatty acids in seeds and pericarp at 18.5% and 11.10%, respectively. Moreover, *L. carinensis* seeds were highly rich in phenolic content. Furthermore, the free radical scavenging action utilizing DPPH results in comparable IC_50_ values with ascorbic acid. Seeds and pericarp displayed also potent α-glucosidase and pancreatic lipase inhibitory activity.^10^ The previous findings have encouraged us for further phytochemical and antiviral investigations of other organs of *L. carinensis*. The current research aimed at exploring the phytochemical metabolome of the plant ethanolic extract. The ultra-high performance liquid chromatography-quadrupole time-of-flight mass spectrometry (UHPLC-QTOF-MS/MS) was applied, which proves its potential abilities for exploration and structural characterization in complex matrices of plants extract particularly that rich in phenolic constituents.^11–13^

In addition, even with advancements in vaccination and medication development, many viruses remain incurable and lack effective vaccinations or antiviral treatments. Hence, discovery of novel antiviral medications is crucial, and natural derived products have proven to be a potential source of such candidates.^14,15^ For instance, the plant extracts of family Aracaceae showed antiviral activities such as the crude extract of *P. dactylifera* fruit pit.^16^ Therefore, the antiviral activity was evaluated for the *L. carinensis* leaves in the scope of phytochemical composition against various viruses, including Adeno (Adeno virus), CoxB4 (Coxsackie B4 virus), HAV (Hepatitis A virus), HSV I (Herpes simplex virus type I) and HSV II (Herpes simplex virus type II) using MTT assay (MTT: 3-[4,5-dimethylthiazol-2-yl]-2,5-diphenyl tetrazolium bromide) using different protocols.^17^ This approach may enable us to address the mechanism of action.

## 2. Material and methods

### 2.1. Plant material and extraction

Leaves of *L. carinensis* (Chiov.) J. Dransf. & N.W. Uhl used in this study were collected in summer of the years 2017–2019 from El-Zoharia and the Orman Botanic Gardens, Egypt. The plants were identified by Mrs Therese Labib, consultant at the Ministry of Agriculture and the former director of the Orman Botanic Garden. A voucher specimen numbered (28 072 019) was archived in the Herbarium of Pharmacognosy Department, Faculty of Pharmacy, Cairo University, Cairo, Egypt.

Following that, 3 kg of air-dried powdered *L. carinensis* leaves were extracted with 70% (v/v) ethanol using cold maceration until exhausted. The ethanolic extracts were collected and evaporated under reduced pressure until dryness, yielding a dry residue of 250 g. The dried residue was then stored in a refrigerator at 4 °C until the following analytical and biological investigations.

### 2.2. UHPLC-QTOF-MS/MS analysis

#### 2.2.1. Sample preparation and analysis parameters

About 50 mg of the dried residue were dissolved in 1 mL of the working solution composed of distilled water : methanol : acetonitrile 50 : 25 : 25. The solubility was enhanced by vortex for 2 min followed 10 min in sonication bath. Then, a centrifugation for 5 min at 10 000 rpm was carried out to remove the insoluble residues. A 20 μL aliquot of the supernatant was diluted by adding 1000 μL of the reconstitution solvent reaching a final concentration at 1 μg μL^−1^. Then, 25 μL were injected for the negative mode analysis against an equal volume of MP-WS as a blank sample.

The sample was analyzed following the protocol of Negm, *et al.* in Proteomics Laboratory of Children Cancer Hospital 57357, Cairo, Egypt.^18^ The instrument consists of ExionLC AC system for chromatographic separation (AB Sciex®, Framingham, MA, USA). The mobile phase consisted of 2 components, where mobile phase A was 5 mM ammonium format buffer pH 8 containing 1% (v/v) methanol, while mobile phase B was 100% acetonitrile. A linear gradient elution was programmed: 0 min, 90% A and 10% B; 21 min, 10% A and 90% B; 25.01 till 28 min, 90% A and 10% B. The column was a pre-column (In-Line filter disks with 0.5 μm × 3.0 mm) purchased from Phenomenex® (Torrance, CA, USA), while the main column type was XBridge C_18_ with 3.5 μm × 2.1 × 50 mm (Waters®, Milford, MA, USA). The column temperature was adjusted at 40 °C with flow rate 0.3 mL min^−1^, and injection volume of 10 μL.

The MS general acquisition information was adjusted for LC-QTOF with a Negative TOF MS (run duration 28 min) using Triple TOF 5600+ system equipped with a Duo-Spray source operating in the ESI mode (AB Sciex®, Concord, ON, Canada). Cycle time (0.6502 s), and no. of cycles (2584). MS1 acquisition was calibrated for TOF Masses (Da) between min = 50.0000; max = 1000.0000, Scan type HR-TOF scan, GS1 was 45, GS2 was 45, CUR was 25, TEM was 500 and ISVF was −4500. In addition, the MS2 acquisition was adjusted for using Information Dependent Acquisition (IDA) as a scan type, with TOF masses (Da) between min = 50.0000–max = 1000.0000, and DP, CE, and CES were 80, −35, and 15, respectively.

#### 2.2.2. Data processing and peak interpretation

The data were processed through MS-DIAL 3.52 which is an open-source software for data-independent acquisition DIA-based identification of small molecules by mass spectral deconvolution. Data-independent acquisition DIA in LC/MS–MS provides comprehensive untargeted acquisition of molecular data. Besides, Respect Negative (1573 recorded compounds) depending on analysis mode was applied, while MasterView was used for feature (peaks) extraction from total ion chromatogram (TIC) based on the following criteria; features should have signal-to-noise greater than 5 (non-targeted analysis), features intensities of the sample-to-blank should be greater than 5 and cutoff score 70% is used to finely select the identified metabolites^18,19^ and consistent with EU Guideline 2002/657/EG.

The metabolic assignments for every peak were determined by utilizing digital natural products databases (METLIN and RIKEN databases) and reference literature to compare retention times and MS data (accurate mass, isotopic distribution, and fragmentation pattern in negative ion mode) of the metabolites detected.

### 2.3. Antiviral assay

The antiviral activity was carried out on the crude ethanolic extract following,^20,21^ where a concentration of 200 mg mL^−1^ of *L. carinensis* leaves extract was prepared using dimethyl sulfoxide (DMSO) (E-Merk). Five different virus isolates were provided by Al-Azhar University, Faculty of Medicine for girls, Microbiology Department, Egypt. They were Adeno (Adeno virus), CoxB4 (Coxsackie B_4_ virus), HAV (Hepatitis A virus), HSV-I (Herpes simplex virus type I), and HSV II (Herpes simplex virus type II). The viral load was for Adeno10^5^, CoxB4 10^5^, HAV 10^4^, HSV I 10^4^, and HSV II 10^4^ copies per mL.

#### 2.3.1. Vero cell

The Vero cell line (*Cercopithecus aethiops*, African Green Monkey, Kidney tissue, Epithelial cells, CCL-81) was purchased from the American Type Culture Collection, USA and maintained in RPMI 1640 medium (Gibco®, Tunisia). The cells were incubated in 5% CO_2_ humidified atmosphere at temperature 37 °C and supplemented with fetal bovine serum (10% v/v), l-glutamine (2 mM), penicillin (100 U mL^−1^) and streptomycin (100 μg mL^−1^).

#### 2.3.2. MTT assay

MTT assay is used to measure cellular metabolism, where the number of live cells present may be reflected by the cellular oxidoreductase enzymes. The tetrazolium dye MTT can be converted by these enzymes to its purple, insoluble formazan. Hence, it is a potential candidate for determination of natural products' cytotoxic activity.^22^ MTT assay is usually done in the dark since MTT reagent is sensitive to light.

The assay was performed following Okba *et al.*,^21^ where the growth medium was decanted from 96 well micro titer plates after confluent sheet formation of Vero cell and the cell monolayer was washed twice with wash medium. Double-fold serial dilutions of tested samples were made in MEM (Minimal Essential Medium). Afterwards, 0.1 mL of each dilution was tested in 3 replicates (*n* = 3) compared with a negative control contained only maintenance medium. The plate was then incubated at 37 °C and checked frequently for two days at the beginning for any physical signs of toxicity, *e.g.*, partial, or complete loss of the monolayer, rounding, shrinkage, or cell granulation. Next, 20 μL of MTT solution prepared in a concentration 5 mg mL^−1^ in PBS were added to each well followed by a step of mixing using a shaking table, 150 rpm for 5 min. At this stage, the reaction was incubated (37 °C, 5% CO_2_) for 1–5 h to allow the metabolism of MTT. The medium of each well was dumped, and the plate was dried on paper towels to remove residue if necessary. The formed formazan was re-suspended in 200 μL DMSO and the plate was placed again on a shaking table to thoroughly mix the formazan into the solvent. Finally, the optical density was recorded at 560 nm and subtracted from the background at 620 nm using a PerkinElmer ELISA reader (HTS 7000 plus). The optical density should be directly correlated with cell quantity. Additionally, the cells were examined every day using a phase-contrast microscope to determine the maximum non-toxic concentration (MNTC) of the extract which was used for the assessment of antiviral activity.

#### 2.3.3. Antiviral protocols

Following,^20,21^ the determined MNTC dilutions of the plant 70% ethanolic extract were tested against the viruses using various protocols as follows: protocol A (virus pretreatment) was recommended to test the virucidal activity of the extract. This was done in order to explore the mechanism of the antiviral activity of the extract. According to this protocol, 100 μL of the combination was injected to the grown Vero cells in 96-well flat-bottom plates after the viruses were exposed to the plant extract for 1 h at 37 °C. After the virus was incubated with Vero cells for 1 h, the plant extract was introduced as part of Protocol B (post infection treatment), which was created to evaluate the extract's impact on virus replication. The third protocol, known as “cell pretreatment” or protocol C, involved incubating a plant extract on Vero cells for 1 h before to exposure to the virus in order to prevent adhesion to the cell surface and evaluate the virus's ability to enter the host cell.

#### 2.3.4. Statistical analysis

Three repetitions were carried out, and the values obtained were expressed as the means of the three independent experiments ± standard error (SE). Student's *t*-test was used to examine differences between a sample and the appropriate control. *p* values less than 0.05 indicated statistical significance for differences, where the significance of the mean differences was calculated using Duncan's multiple range tests.

## 3. Results and discussion

### 3.1. Identification of metabolites

**Fig. 1:**
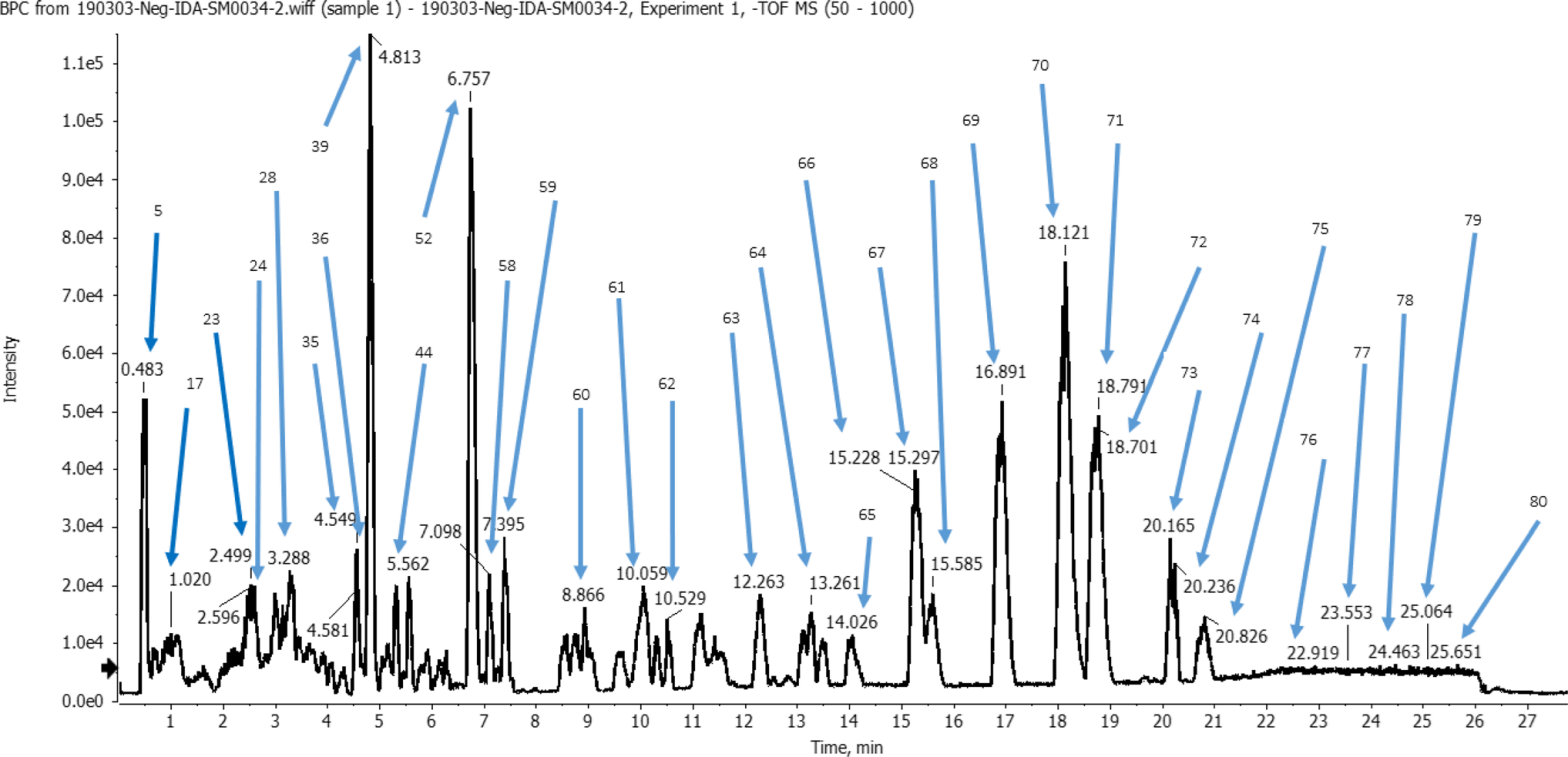
Base peak chromatogram (BPC) of *Livistona carinensis* ethanolic leaves extract in negative mode following analysis by UHPLC-QTOF-MS/MS.

The identification of metabolites was performed in the negative ion mode (ESI), characterized by mass spectra with molecular ions corresponding to [M−H]^−^ and other lower *m*/*z* fragment ions attributed to identification of different classes of metabolites of the plant. A total of 80 peaks were detected from the ethanolic *L. carinensis* leaves extract as shown in the base peak chromatogram, Fig. 1. From which, 72 metabolites were tentatively identified belonging to 14 different classes of metabolites. They were 29 flavonoids, 10 organic acids, 7 phenolic acids, 4 sugars, 9 fatty acids, 2 vitamins, 1 terpene, 1 coumarin, 1 stilbene, 1 lignan, 3 steroids, 1 nucleoside, 1 saponin and 1 amino acid. The results of the analysis are illustrated in Table S1.†

#### 3.1.1. Phenolic acids and derivatives

Various classes of phenolic metabolites were identified such as hydroxybenzoic, hydroxycinnamic, and hydroxyphenylacetic acids in addition to their ester derivatives. Previous reports have revealed that losses of H_2_O and/or CO_2_ were commonly in the MS/MS spectra of phenolic acids.^23^ In the present study, we were able to characterize 7 phenolic acids from *L. carinensis* leaves detected in peaks 7, 8, 17, 20, 26, 29, and 68, Fig. 1.

Hydroxybenzoic acids and derivatives showed a characteristic fragmentation pattern attributed with the detection of the ions at [M–CO_2_] at different retention times due to loss of CO_2_ from their respective precursor ions. When it came to glycoside esters, the characterization was done using neutral loss of glycosidic moiety and fragmentation pattern based on MS data. Moreover, hydroxycinnamic derivatives, particularly, methylated cinnamic acids, showed characteristic demethylated fragment ions [M–CH_3_] and/or [M–CH_3_–H].

#### 3.1.2. Flavonoids

##### 3.1.2.1 Flavonoid aglycone

The presence of six membered rings with double bonds makes a retro-Diels–Alder reaction one of the primary mechanisms for the fragmentation of flavonoid aglycones,^24^ where the resulting fragmentation ions were characteristic. For example, quercetin and its derivatives (*e.g.*, peak 47) were characterized by MS^2^ product ions at *m*/*z* 301 [M–glycoside] or 300 [M–glycoside–H], in addition to characteristic ions at *m*/*z* 179, 151, and 121 were observed, Table S1.† Eriodictyol type and derivatives were also identified. It is a flavanone with molecular ion *m*/*z* 287, which lacks the C ring 2–3 double bond. In the negative mode flavanones were classified into two groups which the spectra were dominated by intense peak at *m*/*z* 151.^24^ Tetrahydroxy flavanone (eriodictyol) was observed in peak 43, Table S1.†

Moreover, kaempferol type and derivatives revealed the presence of peak at *m*/*z* 285, common fragments at *m*/*z* 257 [M–H–CO], *m*/*z* 229 [M–H–2CO], *m*/*z* 211 corresponding to [M–H–H_2_O–2CO], and *m*/*z* 151 as a base peak.^25^ Trihydroxymethoxyflavone (Kaempferide) was detected in peak 54, Table S1.† Luteolin type (Tetrahydroxyflavone) and derivatives were identified in peak 46. These compounds are characteristic by the presence of a base peak at *m*/*z* 285, common fragments at *m*/*z* 175 corresponding to [M–H–2H_2_O–2CO], and peak *m*/*z* 151.^25^ Furthermore, apigenin, tricin, and isorhamnetin type in addition to their derivatives appeared at peaks 51 for apigenin, 35, 44, 52, 59, and 60 for tricin, and 56 for isorhamnetin,^25^ Table S1.†

##### 3.1.2.2 Flavonoid *C*-glycoside

The metabolites of *C*-hexoside compounds showed characteristic fragmentation ions [M–H-90]^−^, [M–H-120]^−^ and [M–H-18]^−^. The higher intensity of [M–H-90] suggests that the attachment of sugar occurs at the 8-position compared with that for 6-glycosides [M–H-120]^−^.^26^ This class of compounds was observed in peaks 14, 21, 22, 27, 31, 57, and 78, Table S1.† Also, *C*-pentosides showed characteristic fragmentation ions [M–H-60]^−^ and [M–H-90]^−^. While in case of di-glycoside, exhibited characteristic two fragmentation ions at (aglycone + 113) and (aglycone + 83).^26^

##### 3.1.2.3 Flavonoid mono-*O*-glycoside

This class of compounds were observed in peaks 13, 15, 24, 25, 30, 32, 38, 40, 42, and 50, which characterized by high abundance of aglycon ion after loss of sugar moiety [M–H-sugar moiety]^−^.

##### 3.1.2.4 Flavonoid-di-*O*-glycoside and *O*-diglycoside

Flavonoid-di-*O*-glucoside was identified in peak 10, which characterized by high abundance of two ions, including the aglycone ion after loss of sugar moiety [M–H-first sugar moiety]^−^ followed by [M–H-di sugar moiety]^−^. While in flavonoid-*O*-diglycoside was observed in peak 35 owing to the high abundance of the aglycon ion after loss of the two sugars moiety [M–H-di sugar moiety]^−^.

#### 3.1.3. Steroids

Three steroids at peaks 58, 72, and 75 were tentatively identified as shown in Fig. 1 and Table S1.† For example, β-sitosterol was identified as β-sitosterol-3-*O*-hexoside (Peak 58). β-Sitosterol has a molecular formula C_29_H_50_O and molecular ion at *m*/*z* 414. Its fragmentation pattern was typical showing *m*/*z* 413 [M−H]^−^, 398 [M–H–CH_3_]^−^, 395 [M–H–H_2_O]^−^, 396 [M–H_2_O]^−^, 380 [M–H–CH_3_–H_2_O]^−^, 328 [M–H–C_6_H_13_]^−^, 302 [M–H–C_7_H_11_O]^−^, 272 [M–H-side chain]^−^, 254 [M–H-side chain–H_2_O]^−^, 230 [M–H-side chain-ring D cleavage–CH_3_]^−^, 212 [M–H-side chain-ring D cleavage–CH_3_–H_2_O]^−^.^27^

#### 3.1.4. Steroidal saponins

Compounds which have the same aglycone skeleton like spirostan saponins having a hexa-cyclic ABCDEF-ring system, have one point of sugar linkage. In all compounds except diosgenin, glycone attachment at C-3 is common with a sugar unit range from 1–4. Diosgenin is an aglycone of spirostan saponins.

The sole steroidal saponin that has been inferred to exist in *L. carinensis* leaves is dioscin (peak 63). In (−)-ESI MS, the chemical yielded a strong deprotonated molecule [M−H]^−^ (*m*/*z* 867.4706). Precursor ion [M+H]^+^ (*m*/*z* 869.4807) in (+)-ESI-MS resulted in 4 fragments (*m*/*z* 723.43, 577.37, 415.32, and 397.31) by the sequential elimination of two rhamnosyl moieties, one glucosyl, and water. It has been reported that the fragment ions at *m*/*z* 271.20 and 253.20 are the diagnostic fragment ions of this kind of steroidal saponins. They resulted from the sequential loss of 144 and 18 Da from the fragment ion at *m*/*z* 415.32. The 18 Da unit was obtained by the loss of a water molecule, while the removal of 144 Da (formula C_8_H_16_O_2_) may have been caused by cleavage of the aglycone's E-ring. In (−)-ESI-MS, diosgenin generated a potent deprotonated molecule [M−H]^−^ (*m*/*z* 413.32). The subsequent loss of 144 and 18 Da is the source of the fragment ions at *m*/*z* 269.20 and 251.20.^28^

#### 3.1.5. Organic acid derivatives

Losses of H_2_O [M–H-18]^−^ and/or CO_2_ [M–H-44]^−^ were regularly observed in the MS/MS spectra of organic acids.^23^*L. carinensis* leaves showed 10 organic acids at peaks 1, 2, 3, 4, 5, 9, 12, 16, 23, and 67 which were tentatively identified, Table S1.†

#### 3.1.6. Terpenes

Terpenes were detected owing to the presence of main fragment ions at *m*/*z* [M–H-18]^−^ due to loss of H_2_O, [M–H-44]^−^ due to loss of CO_2_ indicating the presence of COOH group and [M–H-60]^−^ due to the loss of CH_3_COOH.^29^ One terpene (peak 53) was tentatively identified as gibberellic acid which belongs to diterpene lactones, Table S1.†

#### 3.1.7. Sugars

Monosaccharides were detected owing to the presence of main fragment ions at *m*/*z* [M–H-18]^−^ due to loss of H_2_O, followed by [M–H-18-44]^−^ due to loss of CO_2_ indicating the presence of COOH group. Three monosaccharides were characterized at peaks 19, 37, and 49 and tentatively identified.

#### 3.1.8. Fatty acids

In the second half of the chromatographic run depicted in Fig. 1 at *R*_t_ 12–25 min, MS^*n*^ spectra revealed several saturated and unsaturated fatty acids and derivatives. MS^*n*^ spectra of unsaturated fatty acids derivatives are characterized by product ions at *m*/*z* 277 ([M−H]^−^ octadecatrienoic acid), 279 ([M−H]^−^ octadecadienoic acid), or 281 ([M−H]^−^ octadecenoic acid). Five unsaturated fatty acids at peaks 62, 64, 69, 70, and 77 were tentatively identified. In addition, four saturated fatty acids were characterized at peaks 36, 55, 74, and 76 and tentatively identified.

#### 3.1.9. Miscellaneous metabolites

Two vitamins (peaks 65 and 66), 1 stilbene (peak 41), 1 lignan (peak 28), 1 amino acid (peak 6), 1 coumarin (peak 18), and 1 nucleoside (peak 11) were tentatively identified in the leaves ethanolic extract of *L. carinensis*, Table S1.†

### 3.2. Antiviral activities

#### 3.2.1. Cytotoxicity assay

The MNTC for *L. carinensis* was found to be 156.25 μg mL^−1^ and the CC_50_ was found to be 790.6 μg mL^−1^, Table 1.

**Table 1:** Determination of *L. carinensis* samples cytotoxicity on Vero cells (*n* = 3)^a^

ID	Dilution	Mean O.D ± SE	Viability%	Toxicity%	CC_50_
Vero	μg mL^−1^	0.247 ± 0.0012	100	0	μg mL^−1^
*L. carinensis*	10 000	0.022 ± 0.0009	9.0418	90.9582	790.68
	5000	0.024 ± 0.0023	9.5816	90.4184	
	2500	0.028 ± 0.0012	11.3360	88.6640	
	1250	0.055 ± 0.0047	22.2672	77.7328	
	625	0.138 ± 0.0015	56.0054	43.9946	
	312.5	0.195 ± 0.0045	78.9474	21.0526	
	156.25	0.234 ± 0.0035	94.7368	5.2632	
	78.12	0.247 ± 0.0017	100	0	

a O.D: optical density S.E.: standard error; CC_50_: 50% cytotoxic concentration, defined as the compound's concentration required for the reduction of cells viability by 50%.

#### 3.2.2. Antiviral assay

##### 3.2.2.1 - Protocol A (anti-replicative)

*L. carinensis* extract showed powerful antiviral activity (58.6%) against Adeno virus following Protocol A, Table 2. In addition, the bigger the SI value, the more antiviral activity, SI value of *L. carinensis* was (5.9) antiviral activity against Adeno virus is higher than that of acyclovir (0.2). Moreover, *L. carinensis* extract has greater antiviral activity (38.9%) with SI (3.9) against Coxsackie B4 virus compared to acyclovir (30.3%) with SI = 1.9. In addition, it showed powerful antiviral activity (68.3%) with SI (6.9) against Hepatitis A virus compared to acyclovir (25.5%) with SI (1.6). *L. carinensis* extract (40.9%) with SI (4.1), compared to acyclovir which showed good activity (59.0%) with SI (3.80). However, *L. carinensis* extract (26.53%) showed weak antiviral activity (3.07%) compared to acyclovir (83.2%) with SI (5.32) against HSV-I. Table 3 describes the antiviral activity of *L. carinensis* extract compared to acyclovir.

**Table 2:** Antiviral activity of *L. carinensis* extract using different protocols for five viruses (*n* = 3)^a^

Name of the virus	Measured parameters	Protocol A	Protocol B	Protocol C
Adeno virus (adeno)	Mean O.D ± S.E	0.1843 ± 0.0049	0.1113 ± 0.0012	0.1463 ± 0.0034
	Antiviral activity% (M ± S.E)	58.610 ± 0.651	4.494 ± 0.053	28.090 ± 0.421
	IC_50_ (μg mL^−1^)	133.29	—	278.12
	S.I	5.9317	0.4549	2.843
	%RSD	4.605	1.867	4.025
Coxsakie B4 (CoxB4)	Mean O.D ± S.E	0.1447 ± 0.0035	0.1343 ± 0.0046	0.0940 ± 0.0044
	Antiviral activity% (M ± S.E)	38.902 ± 0.816	29.163 ± 0.495	5.169 ± 0.129
	IC_50_ (μg mL^−1^)	200.82	267.88	1511.41
	S.I	3.9371	2.9515	0.523
	%RSD	4.189	5.932	8.107
Hepatitis a virus (HAV)	Mean O.D ± S.E	0.1907 ± 0.002404	0.1627 ± 0.0046	0.1687 ± 0.0041
	Antiviral activity% (M ± S.E)	68.279 ± 1.570	36.235 ± 0.688	40.471 ± 0.890
	IC_50_ (μg mL^−1^)	114.42	215.60	193.04
	S.I	6.9103	3.6673	4.096
	%RSD	2.183	4.897	4.209
Herpes simplex virus type I (HSV I)	Mean O.D ± S.E	0.1423 ± 0.0043	0.1453 ± 0.0047	0.1220 ± 0.0031
	Antiviral activity% (M ± S.E)	26.536 ± 0.613	26.757 ± 0.768	10.884 ± 0.261
	IC_50_ (μg mL^−1^)	294.41	291.9753	717.77
	S.I	2.6856	2.7080	1.102
	%RSD	5.233	5.603	4.401
Herpes simplex virus type II (HSV II)	Mean O.D ± S.E	0.1767 ± 0.0049	0.1357 ± 0.0026	0.1697 ± 0.0044
	Antiviral activity% (M ± S.E)	40.959 ± 0.901	1.401 ± 0.042	29.972 ± 0.569
	IC_50_ (μg mL^−1^)	190.73	—	260.66
	S.I	4.1453	0.1418	3.033
	%RSD	4.803	3.318	4.491

a S.I (selectivity index) CC_50_/IC_5_; the ratio between the cytotoxic and the antiviral activity of a tested sample (CC_50_ = 790.68 (μg mL^−1^)). % RSD: % Relative standard deviation for determinations corresponds to viruses in each protocol.

**Table 3:** % Antiviral activity of *L. carinensis* extract compared to Acyclovir. The results are represented as % mean ± S.E. (*n* = 3)

Virus name	Plant extract	Protocol A antiviral%	Protocol B antiviral%	Protocol C antiviral%
Adeno virus	*L. carinensis*	58.610 ± 0.651	4.494 ± 0.053	28.090 ± 0.421
	Acyclovir	2.710 ± 0.054	38.644 ± 0.618	63.343 ± 1.203
CoxB4 virus	*L. carinensis*	38.902 ± 0.816	29.163 ± 0.495	5.169 ± 0.129
	Acyclovir	30.310 ± 0.484	28.326 ± 0.538	63.491 ± 0.761
HAV	*L. carinensis*	68.279 ± 1.570	36.235 ± 0.688	40.471 ± 0.890
	Acyclovir	25.537 ± 0.745	2.286 ± 0.529	27.529 ± 0.621
HSV I	*L. carinensis*	26.536 ± 0.613	26.757 ± 0.768	10.884 ± 0.261
	Acyclovir	83.240 ± 5.67	54.666 ± 1.32	80.287 ± 2.62
HSV II	*L. carinensis*	40.959 ± 0.901	1.401 ± 0.042	29.972 ± 0.569
	Acyclovir	59.409 ± 1.128	39.258 ± 0.628	48.016 ± 0.963

##### 3.2.2.2 - Protocol B (anti-infective)

*L. carinensis* extract showed weak (4.5%) antiviral activity against Adeno virus compared to acyclovir (38.6%). Similarly, it had a weak activity against HSV I and HSV II at 26.8% and 1.4%, and 1.68% compared to acyclovir compared to acyclovir at 54.7% and 39.25%, respectively. However, it showed greater activity (29.2%) with SI (2.95) against Coxsackie B4 virus than acyclovir (28.3%) with SI (1.8), Tables 2 and 3. Thus, the tested extract showed mostly weak antiviral activity against all tested viruses, compared to acyclovir as reference drug.

##### 3.2.2.3 Protocol C (antiviral)

*L. carinensis* extract showed weak antiviral activity against Adeno virus (28.1% *vs.* 63.3% for acyclovir), Hepatitis A virus (40.5%) with SI (4.1 *vs.* 27.5% for acyclovir) and HSV-II (29.9% *vs.* 48.0% for acyclovir): Also, it showed weak effect against Coxsakie B4 (CoxB4) virus (5.2% *vs.* 63.5%) and HSV-I (10.88% *vs.* 80.28% for acyclovir), Tables 2 and 3.

Despite of the high % antiviral inhibition of acyclovir, it needs high doses to be effective when consumed orally, that can cause many side effects among individuals with serious illness and frequent toxic effects.^30^ Following Protocol A (anti-replicative), *L. carinensis* extract showed powerful antiviral activity (58.6%) against Adeno virus, antiviral activity (38.9%) against Coxsackie B4, and against HCV (68.27%) compared to acyclovir (2.71%, 30.3% and 25.5%, respectively). Because of this activity, it can be used as antiviral supplement in primitive areas and where the synthetic antiviral drugs hard to be found. However, *L. carinensis* extract showed (26.5% and 40.95%) weak antiviral activity compared to acyclovir (83.2% and 59.4%) against HSV-I and HSV II, Table 3.

## 4. Conclusions and future prospective

The current study can be considered novel of its kind, where none of the previous studies have investigated the phytochemical composition and antiviral potential of *L. cainensis* leaves. A comprehensive phytochemical investigation its ethanolic extract has resulted in a tentative identification of 72 metabolites belonging to 14 classes. Hence, the present study is in agreement with previous studies showing the richness of Aracaceae in phenolic compounds. The results were also consistent with the chemical results proving its candidacy to possess potential antiviral effects compared with acyclovir. Moreover, *L. carinensis* extract showed potent antiviral activity against HAV using the three protocols tested compared to acyclovir as reference drug, while it showed a significant antiviral activity against Adeno virus using protocol A only, which means it causes direct virus inactivation rather than inhibition of virus replication or blocking its attachment to Vero cells. *L. carinensis* extract also showed moderate activity against CoxB4 virus by direct virus inactivation and inhibition of its replication, compared to acyclovir as reference standard drug. Beside studying the antiviral activity of crude ethanolic extract of the leaves, it is highly recommended to work on further fractionation and isolation of major metabolites to support the UHPLC/LC-MS tentative identification. NMR is a conclusive tool that can support and confirm these findings. These further investigations may help check the antiviral activity of resulted fractions (*e.g.*, ethyl acetate and n-butanol fractions) that show the major phytochemicals according to TLC, in addition to the isolated metabolites, and consequently the possible molecular mechanisms.

## Author contributions

All authors contribute equally to this work and have read and agreed to the published version of the manuscript.

## Conflicts of interest

The authors report no conflict of interest.

## Supplementary Material

RA-014-D4RA02705A-s001
