# Supplementary material for: Phytochemical profiling of Livistona carinensis leaf extract via UHPLC-QTOF-MS/MS with assessment of its antiviral mechanisms
Source: RSC Adv. 2024 Jul 5;14(30):21300–6. doi: 10.1039/d4ra02705a (PMC11225548; doi:10.1039/d4ra02705a)
Supplement: RA-014-D4RA02705A-s001 [file RA-014-D4RA02705A-s001.pdf]

Electronic Supplementary Material (ESI) for RSC Advances.
This journal is © The Royal Society of Chemistry 2024

Supplementary Data

Phytochemical profiling of *Livistona carinensis* leaves extract *via* UHPLC-QTOF-MS/MS and NMR with assessment of its antiviral mechanisms

Amr M.K. Mahrous[1*], Mohamed S. Hifnawy[2], Rehab M.S. Ashour[2], Marwa Yousry Issa[2], Ahmed Zayed[3*]

[1] Faculty of Pharmacy, El Saleheya El Gadida University, El Saleheya El Gadida 44813, Egypt

[2] Department of Pharmacognosy, Faculty of Pharmacy, Cairo University, Kasr El-Aini Street, Cairo 11562, Egypt

[3] Department of Pharmacognosy, College of Pharmacy, Tanta University, El-Guish Street (Medical Campus), 31527 Tanta, Egypt

*** Correspondence**

- **Dr. Amr M.K. Mahrous**

  Faculty of Pharmacy, El Saleheya El Gadida University, El Saleheya El Gadida 44813, Egypt

  E-Mail: amrmkh@gmail.com

- **Dr. Ahmed Zayed**

  Department of Pharmacognosy, College of Pharmacy, Tanta University, El-Guish Street (Medical Campus), 31527 Tanta, Egypt

  E-Mail: ahmed.zayed1@pharm.tanta.edu.eg

Table S1. Metabolites detected in the ethanolic extract of *Livistona carinensis* leaves *via* UPLC-QTOF-MS/MS

Peak No.	Rt (min)	Precursor ion $[M-H]^-$	Elemental composition	Base peak	Error (ppm)	MS/MS fragments	Metabolites assignment	Chemical class	Ref.
1	0.4300	173.0086	$C_6H_6O_6$	173	0.0098	173, 129, 111, 85	Aconitic acid	Organic acid	[1]
2	0.4657	191.0207	$C_6 H_8 O_7$	111	0.0203	191, 173, 129, 111, 85	Citric acid	Organic acid	[2]
3	0.4658	133.0142	$C_4H_6O_5$	133	0.0145	133, 115, 71	Malic acid	Organic acid	[3]
4	0.4659	117.0196	$C_4H_6O_4$	73	0.0182	117, 73	Succinic acid	Organic acid	[4]
5	0.4800	147.0301	$C_5H_8O_5$	129	0.0302	147, 129, 85, 59	Citramalic acid	Organic acid	[5]
6	0.4900	128.0361	$C_5H_7NO_3$	128	0.0358	128, 127, 82	Oxo-proline	Amino acid derivative	[6]
7	0.5000	179.0577	$C_9H_8O_4$	135	0.0563	179, 161, 135, 131, 89, 85, 71, 59	Caffeic acid	Phenolic acid	[7]
8	0.5172	353.0893	$C_{16}H_{18}O_9$	191	0.0885	353, 191, 179	Chlorogenic acid	Phenolic acid	[6]
9	0.5172	137.0240	$C_6H_6N_2O_2$	93	0.0251	137, 93, 79, 65	Urocanic acid	Organic acid derivative	[8]
10	0.5301	609.1396	$C_{27}H_{30}O_{16}$	449	0.0615	609, 447, 285	Luteolin-di-O-hexose	Flavone di-glycoside	[9]
11	0.5560	134.0473	$C_5H_5N_5$	134	0.0475	134, 107, 92	Adenine	Nucleoside	[6]
12	0.6200	175.0598	$C_7H_{12}O_5$	175	0.0618	175, 157, 131, 115, 113, 85, 73	Isopropylmalic acid	Organic acid	[10]

13	0.7330	461.1281	$C_{21}H_{18}O_{12}$	461	0.1318	461, 419, 285, 193, 151	Kaempferol-hexuronide	Flavonoid glycoside	[11]
14	0.7340	433.1396	$C_{25}H_{22}O_7$	433	0.1351	433, 395, 300, 179	Haplopappin	Flavonoid	[10]
15	0.8250	447.1506	$C_{21}H_{20}O_{11}$	447	0.1524	447, 429, 401, 357, 327, 299, 293	Kaempferol-*O*-hexoside	Flavonoid glycoside	[9]
16	0.9100	130.9833	$C_5H_8O_4$	87	0.9835	131, 87, 57	Methylsuccinic acid	Organic acid derivative	[8]
17	1.0200	137.0257	$C_7H_6O_3$	137	0.0249	137, 136, 108, 93	Salicylic acid	Phenolic acid derivative	[1]
18	1.4060	177.0211	$C_9H_6O_4$	177	0.0201	177, 149, 133	Dihydroxy-coumarin (Aesculetin)	Hydroxy-coumarin	[6]
19	1.4061	343.1026	$C_{12}H_{24}O_{11}$	343	0.1050	343, 299, 137, 89	Maltitol	Disaccharide	[11]
20	2.0210	385.1860	$C_{17}H_{22}O_{10}$	385	0.1884	385, 325, 223, 152, 89	Sinapoyl hexoside	Hydroxy-cinnamic acid glycoside	[10]
21	2.0380	431.1924	$C_{21}H_{20}O_{10}$	431	0.1927	431, 341, 311, 283, 269	Apigenin-*C*-hexoside (Vitexin)	Flavone glycoside	[9]
22	2.0382	447.0934	$C_{21}H_{20}O_{11}$	447	0.0956	447, 401, 357, 327, 297, 285	Luteolin-C-hexoside (Isoorientin)	Flavonoid glycosides	[12]
23	2.4990	60.9929	CH_2O_3	60.99	0.0229	60.99	Carbonic acid	Organic acid	[1]
24	2.5960	593.1507	$C_{27}H_{30}O_{15}$	593	0.1507	593, 473, 413, 315	Isorhamnetin-O-pentoside deoxyhexose	Flavonoid glycoside	[9]

25	2.7980	593.1524	$C_{27}H_{30}O_{15}$	593	0.1515	593, 431, 413, 293	Kaempferol-O-hexose-deoxyhexoside	Flavonoid glycosides	9
26	2.8440	167.0343	$C_8H_8O_4$	167	0.0346	167, 152, 124, 108	Homogentisic acid	Phenolic acid	6
27	3.1390	431.0990	$C_{21}H_{20}O_{10}$	431	0.0998	431, 341, 311, 283	Apigenin-C-hexoside (Vitxein)	Flavone glycoside	12
28	3.2900	523.2192	$C_{26}H_{36}O_{11}$	361	0.1232	523, 492, 477, 361	Secoisolariciresinol mono-hexoside	Lignan	10
29	3.4400	138.0193	$C_6H_5NO_3$	138	0.0199	138, 108, 92	Para-nitrophenol	Phenolics	6
30	4.0900	623.1631	$C_{28}H_{32}O_{16}$	623	0.1641	623, 577, 315	Isorhamnetin-*O*-hexose-deoxyhexoside (Narcissin)	Flavonol glycoside	11
31	4.2100	447.0939	$C_{21}H_{20}O_{11}$	447	0.0941	447, 357, 327, 297, 285, 137	Luteolin-C-hexose (Orientin)	Flavone glycoside	10
32	4.2900	507.1152	$C_{23}H_{24}O_{13}$	507	0.1139	507, 461, 345	Syringetin-*O*-hexoside	Flavonol glycoside	10
33	4.3100	449.1508	$C_{21}H_{22}O_{11}$	449	0.1491	449, 287	Okanin-*O*-hexoside (Marein)	Chalcone/ Aurone	10
34	4.5091	341.0676	$C_{12}H_{22}O_{11}$	341	0.0677	341, 179, 161	Dihexose	Disaccharide	8
35	4.5490	637.1762	$C_{29}H_{34}O_{16}$	329	0.0659	637, 329, 314	Tricin-*O*-hexose-deoxyhexoside	Flavone glycoside	9
36	4.5810	317.1498	$C_{16}H_{31}BrO$	225	0.1142	317, 299, 225, 207, 181	Bromopalmitaldehyde	Fatty acid derivative	10
37	4.7060	193.0517	$C_6H_{10}O_7$	193	0.0508	193, 178, 161, 133	Uronic acid	Sugar acid	13

38	4.7070	477.1378	$C_{22}H_{22}O_{12}$	477	0.1439	477, 315, 271	Isorhamnetin-*O*-hexoside	Flavonol glycoside	[14]
39	4.8130	377.1758	$C_{24}H_{26}O_{4}$	241	0.0174	377, 345, 241, 152, 137	Albafuran A	Benzofuran	[6]
40	4.8347	507.1468	$C_{23}H_{24}O_{13}$	507	0.1493	507, 345	Syringetin-*O*-hexoside	Flavonol glycoside	[10]
41	4.8471	405.1709	$C_{20}H_{22}O_{9}$	405	0.1683	405, 243	Trihydroxy-3'-hexopyranosylstilbene (Astringin)	Stilbene glycoside	[6]
42	5.0495	445.1853	$C_{21}H_{18}O_{11}$	269	0.1860	445, 409, 269, 161	Baicalein-*O*-hexouronide (Baicalin)	Flavone glycoside	[11]
43	5.1845	287.0564	$C_{15}H_{12}O_{6}$	287	0.0565	287, 151, 135, 134	Tetrahydroxy flavanone (Eriodictyol)	Flavanone	[15]
44	5.5620	869.2329	$C_{54}H_{77}O_{9}$	869	0.1786	869, 833, 637, 329	Tricin derivative	------	------
45	5.5645	611.1412	$C_{28}H_{36}O_{15}$	611	0.1452	611, 565, 413, 311	Neohesperidin dihydrochalcone	Chalcone/ Aurone	[6]
46	5.6345	285.0411	$C_{15}H_{10}O_{6}$	285	0.0409	285, 241, 175, 151, 133	Tetrahydroxyflavone (Luteolin)	Flavones	[9]
47	5.6855	301.0340	$C_{15}H_{10}O_{7}$	301	0.0361	301, 273, 255, 193, 179, 151, 121	Pentahydroxyflavone (Quercetin)	Flavonol	[12]
48	5.8207	449.1473	$C_{21}H_{22}O_{11}$	287	0.1451	449, 413, 341, 287	Marein (Okanin-*O*-hexose)	Chalcone/ Aurone	[10]
49	5.9246	195.1016	$C_6H_{12}O_7$	195	0.1025	195, 151, 123, 94	Pentahydroxyhexanoic acid (Gluconic acid)	Sugar acid	[8]

50	6.3963	433.1507	$C_{21}H_{22}O_{10}$	217	0.1523	433, 271, 256, 241	Naringenin-*O*-hexoside (Prunin)	Flavanone glycoside	10
51	6.5200	269.0465	$C_{15}H_{10}O_{5}$	269	0.0467	269, 151, 117	Trihydroxyflavone (Apigenin)	Flavone	16
52	6.7570	329.0676	$C_{17}H_{14}O_{7}$	329	0.0659	329, 314, 299, 271	Tricin	Flavone	6
53	6.7828	345.059	$C_{19}H_{22}O_{6}$	345	0.0632	345, 330, 315, 307, 285, 137	Gibberellic acid	Diterpene lactones	17
54	6.7951	299.0553	$C_{16}H_{12}O_{6}$	299	0.0567	299, 285, 284, 151	Trihydroxymethoxyflavone (Kaempferide)	Flavonol	16
55	6.8601	381.1511	$C_{19}H_{26}O_{8}$	381	0.1552	381, 251, 87	Myristoyl-hydroxy-sn-glycerol	Fatty acid derivative	18
56	7.0754	315.0526	$C_{16}H_{12}O_{7}$	315	0.0514	315, 300, 269, 79	Tetrahydroxymethoxyflavone (Isorhamnetin)	Flavonol	16
57	7.0883	415.1511	$C_{21}H_{20}O_{9}$	415	0.1512	415, 295	Daidzein-*C*-hexoside (Puerarin)	Isoflavone *C*-glycoside	9
58	7.0980	575.4317	$C_{35}H_{60}O_{6}$	575	0.1407	575, 413, 363	*β*-Sitosterol-3-*O*-hexoside	Steroid	19
59	7.3950	588.1413	$C_{16}H_{25}N_{5}O_{15}$	588	0.1380	588, 525, 329, 314	Tricin derivative	Flavonoid glycoside	9
60	8.8660	711.1517	$C_{31}H_{36}O_{19}$	675	0.1720	711, 675, 329, 137	Tricin derivative	Flavonoid glycoside	9
61	10.059	645.2010	$C_{32}H_{38}O_{14}$	645	0.1969	645, 525, 315, 270, 137	Isorhamnetin derivative	Flavonoid glycoside	6
62	10.5290	459.3143	$C_{31}H_{56}O_{2}$	459	0.3111	459, 415	Pentacosyl-1,3-benzenediol	Fatty acid derivative	20

63	12.2630	867.4821	$C_{45}H_{72}O_{16}$	867	0.2291	867, 413, 329	Dioscin	Steroidal saponin	[19]
64	13.2610	639.2294	$C_{25}H_{49}O_{11}P$	555	0.2823	639, 555, 225	Hexadecenyl-glycero-3-phospho-(1'-myo-inositol)	Fatty acid derivative	[6]
65	14.0260	453.3028	C29H42 O_4	453	0.2980	453, 409, 407, 339	Epoxy-tetradehydro-dihomo-dihydroxycholecalciferol	Vitamin D derivative	[10]
66	15.2280	425.3282	$C_{29}H_{46}O_2$	425	0.2506	425, 381, 139	hydroxy-24-methylergocalciferol	Vitamin D derivative	[10]
67	15.2970	89.0261	$C_3H_6O_3$	89	0.0261	89, 71	Dihydroxyacetone	Organic acid	[1]
68	15.5850	369.1107	$C_{17}H_{22}O_9$	277	0.2192	368, 306, 277	Sinapaldehyde hexoside	Phenolic glycoside	[9]
69	16.8910	559.4199	$C_{36}H_{64}O_4$	279	0.2333	559, 279	Linoleic acid dimer	Fatty acid derivative	[20]
70	18.1210	585.5093	$C_{36}H_{58}O_6$	255	0.2337	585, 255	Deoxyphorbol-palmitate	Fatty acid derivative	[20]
71	18.7010	685.5196	$C_{31}H_{42}O_{17}$	685	0.2455	685, 649, 365, 281	Unknown	---	---
72	18.7910	401.2425	$C_{24}H_{34}O_5$	281	0.2461	401, 325, 281, 137	Trioxo-cholanoic acid	Steroids	[19]
73	20.1650	701.4745	$C_{31}H_{42}O_{18}$	701	0.4745	701, 569, 419, 302, 119	Unknown	---	---
74	20.2360	199.1741	$C_{12}H_{24}O_2$	129	0.1741	199, 147, 127, 69, 59	Methyl-undecanoic acid	Fatty acid derivative	[20]
75	20.8260	359.1939	$C_{21}H_{28}O_5$	359	0.1939	359, 285, 283, 135	Dihydroprednisone	Steroids	[19]

76	22.9190	461.3570	$C_{25}H_{50}O_7$	461	0.3446	461, 193, 151, 138	Hexopyranosyl-1,2-nonadecandiol	Fatty acyl glycoside	[20]
77	23.5530	461.3664	$C_{31}H_{58}O_2$	461	0.3641	461, 419, 311, 293, 195, 151	Hentriacontene-2,4-dione	Fatty acid derivative	[20]
78	24.4630	447.0945	$C_{21}H_{20}O_{11}$	447	0.3753	447, 357, 327, 285, 177	Luteolin-*C*-hexoside	Flavonoid glycoside	[9]
79	25.0640	667.5442	$C_{43}H_{72}O_5$	665	0.5374	665, 377, 283, 136, 62	Unknown	---	---
80	25.6510	665.5089	$C_{43}H_{70}O_5$	62	0.5089	665, 377, 283, 245, 212, 138 136, 62	Unknown	---	---

References

1. M. Boğa, A. Ertaş, M. A. Yılmaz, M. Kızıl, B. Çeken, N. Haşimi, T. Y. Özden, S. Demirci, İ. Yener and Ö. Deveci, *Iranian journal of pharmaceutical research : IJPR*, 2016, **15**, 393-405.
2. O. Al Kadhi, A. Melchini, R. Mithen and S. Saha, *Journal of analytical methods in chemistry*, 2017, **2017**, 5391832.
3. A. D. Watson, *Journal of Lipid Research*, 2006, **47**, 2101-2111.
4. L. Tretter, A. Patocs and C. Chinopoulos, *Biochimica et Biophysica Acta (BBA) - Bioenergetics*, 2016, **1857**, 1086-1101.
5. W. Shaw, E. Kassen and E. Chaves, *Clinical chemistry*, 1995, **41**, 1094-1104.
6. I. M. Abu-Reidah, M. M. Contreras, D. Arráez-Román, A. Segura-Carretero and A. Fernández-Gutiérrez, *Journal of chromatography. A*, 2013, **1313**, 212-227.
7. R. Ahmed, E. Elkhrisy, W. El-kashak, M. El-Raey, M. Nassar and E.-S. Aboutabl, *Journal of Advanced Pharmacy Research*, 2019, **3**, 23-29.
8. H. Horai, M. Arita, S. Kanaya, Y. Nihei, T. Ikeda, K. Suwa, Y. Ojima, K. Tanaka, S. Tanaka, K. Aoshima, Y. Oda, Y. Kakazu, M. Kusano, T. Tohge, F. Matsuda, Y. Sawada, M. Y. Hirai, H. Nakanishi, K. Ikeda, N. Akimoto, T. Maoka, H. Takahashi, T. Ara, N. Sakurai, H. Suzuki, D. Shibata, S. Neumann, T. Iida, K. Tanaka, K. Funatsu, F. Matsuura, T. Soga, R. Taguchi, K. Saito and T. Nishioka, *Journal of mass spectrometry : JMS*, 2010, **45**, 703-714.
9. I. M. Abu-Reidah, M. S. Ali-Shtayeh, R. M. Jamous, D. Arráez-Román and A. Segura-Carretero, *Food Chemistry*, 2015, **166**, 179-191.
10. Y. Sawada, K. Akiyama, A. Sakata, A. Kuwahara, H. Otsuki, T. Sakurai, K. Saito and M. Y. Hirai, *Plant & cell physiology*, 2009, **50**, 37-47.
11. F. Allen, R. Greiner and D. Wishart, *Metabolomics*, 2015, **11**, 98-110.
12. F. Sánchez-Rabaneda, O. Jáuregui, I. Casals, C. Andrés-Lacueva, M. Izquierdo-Pulido and R. M. Lamuela-Raventós, *Journal of mass spectrometry : JMS*, 2003, **38**, 35-42.
13. F. Matsuda, R. Nakabayashi, Y. Sawada, M. Suzuki, M. Y. Hirai, S. Kanaya and K. Saito, *Frontiers in plant science*, 2011, **2**, 40.
14. I. Parejo, O. Jauregui, F. Sánchez-Rabaneda, F. Viladomat, J. Bastida and C. Codina, *Journal of agricultural and food chemistry*, 2004, **52**, 3679-3687.
15. N. Fabre, I. Rustan, E. de Hoffmann and J. Quetin-Leclercq, *Journal of the American Society for Mass Spectrometry*, 2001, **12**, 707-715.
16. A. Bouhafsoun, M. Yilmaz, A. Boukeloua, H. Temel and M. Kaid-Harche, *Ciência e Tecnologia de Alimentos*, 2018, **38**.
17. N. Takeda, Y. Handa, S. Tsuzuki, M. Kojima, H. Sakakibara and M. Kawaguchi, *Plant Signaling & Behavior*, 2015, **10**, e1028706.
18. A. Moritz, P. N. De Graan, W. H. Gispen and K. W. Wirtz, *The Journal of biological chemistry*, 1992, **267**, 7207-7210.
19. B. Avula, Y. H. Wang, M. Wang, Z. Ali, T. J. Smillie, J. Zweigenbaum and I. A. Khan, *Planta medica*, 2014, **80**, 321-329.
20. A. M. Murad, G. R. Vianna, A. M. Machado, N. B. da Cunha, C. M. Coelho, V. A. Lacerda, M. C. Coelho and E. L. Rech, *Analytical and bioanalytical chemistry*, 2014, **406**, 2873-2883.
21. E. Stahl, in *Thin-Layer Chromatography: A Laboratory Handbook*, Springer, 1969, pp. 52-86.
22. A. Asami, Y. Hiral and J. Shoji, *Chemical and pharmaceutical bulletin*, 1991, **39**, 2053-2056.
23. S. Zona, *Harvard Papers in Botany*, 1997, **2**, 71-107.
24. K. R. Markham, *Techniques of flavonoid identification*, 1982.
25. T. J. Mabry, K. Markham, M. Thomas, T. J. Mabry, K. Markham and M. Thomas, *Journal*, 1970, 41-146.
26. Z. Güvenalp, H. Özbek, T. Ünsalar, C. Kazaz and L. Demirezer, *Turkish Journal of Chemistry*, 2006, **30**, 391-400.

27. H. Wagner, S. Bladt and E.-M. Zgainski, *Drogenanalyse: dünnschichtchromatographische analyse von arzneidrogen*, Springer-Verlag, 2013.
28. J. Goad and T. Akihisa, *Analysis of sterols*, Springer Science & Business Media, 2012.
29. H. Ibrahim, F. Elshaarawy and E. Haggag, *Journal of Advanced Pharmacy Research*, 2018, **2**, 256-268.
30. D.-M. Wang, W.-J. Pu, Y.-H. Wang, Y.-J. Zhang and S.-S. Wang, *Molecules (Basel, Switzerland)*, 2012, **17**, 4595-4603.
31. S. Prinz, A. Ringl, A. Huefner, E. Pemp and B. Kopp, *Chemistry & Biodiversity*, 2007, **4**, 2920-2931.
32. K. Idaka, Y. Hirai and J. Shoji, *Chemical and pharmaceutical bulletin*, 1991, **39**, 1455-1461.
